# [*meso*-5,10,15,20-Tetra­kis(5-bromo­thio­phen-2-yl)porphyrinato-κ^4^ *N*,*N*′,*N*′′,*N*′′′]nickel(II)

**DOI:** 10.1107/S1600536812011671

**Published:** 2012-03-24

**Authors:** R. Prasath, P. Bhavana, Seik Weng Ng, Edward R. T. Tiekink

**Affiliations:** a Department of Chemistry, BITS, Pilani – K. K. Birla Goa Campus, Goa 403 726, India; b Department of Chemistry, University of Malaya, 50603 Kuala Lumpur, Malaysia; c Chemistry Department, Faculty of Science, King Abdulaziz University, PO Box 80203 Jeddah, Saudi Arabia

## Abstract

The Ni^II^ atom in the title porphyrin complex, [Ni(C_36_H_16_Br_4_N_4_S_4_)], is in a square-planar geometry defined by four pyrrole N atoms. There is considerable buckling in the porphyrin ring with the dihedral angles between the N_4_ donor set and the pyrrole rings being in the range 17.0 (3)–18.8 (3)°. Each of the six-membered chelate rings is twisted about an Ni—N bond and the dihedral angles between diagonally opposite chelate rings are 13.08 (15) and 13.45 (11)°; each pair of rings is orientated in opposite directions. The bromo­thienyl rings are twisted out of the plane of the central N_4_ core with dihedral angles in the range 51.7 (2)–74.65 (19)°. Supra­molecular chains along [001] are formed through C—H⋯Br inter­actions in the crystal packing. Three of the four bromo­thienyl units are disordered over two coplanar positions of opposite orientation with the major components being in 0.691 (3), 0.738 (3) and 0.929 (9) fractions.

## Related literature

For general background and potential applications of thienyl porphyrins, see: Boyle *et al.* (2010 ▶); Chen *et al.* (2010 ▶); Paul-Roth *et al.* (2008 ▶); Rochford *et al.* (2008 ▶); Wallin *et al.* (2006 ▶); Friedlein *et al.* (2005 ▶); Bhyrappa & Bhavana (2001 ▶). For related structures, see: Ghazzali *et al.* (2008 ▶); Bhyrappa *et al.* (2006 ▶); Purushothaman *et al.* (2001 ▶).
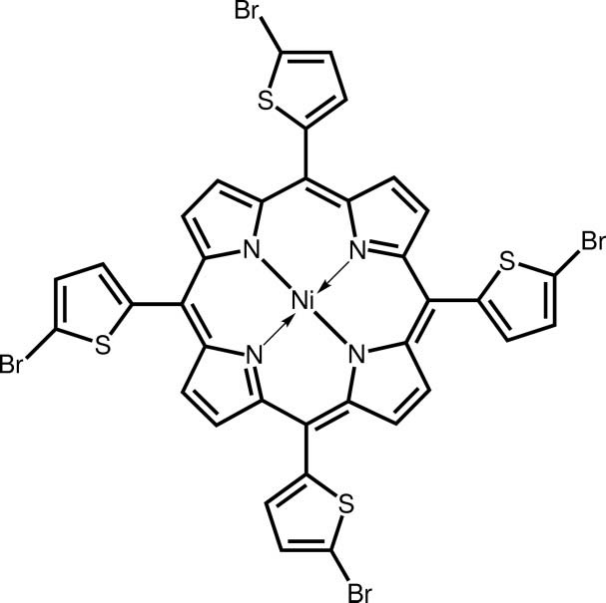


## Experimental

### 

#### Crystal data

[Ni(C_36_H_16_Br_4_N_4_S_4_)]
*M* *_r_* = 1011.12
Orthorhombic, 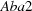

*a* = 21.9367 (9) Å
*b* = 19.0090 (9) Å
*c* = 16.1742 (6) Å
*V* = 6744.6 (5) Å^3^
*Z* = 8
Mo *K*α radiation
μ = 5.60 mm^−1^
*T* = 100 K
0.30 × 0.25 × 0.20 mm

#### Data collection

Agilent SuperNova Dual diffractometer with an Atlas detector
Absorption correction: multi-scan (*CrysAlis PRO*; Agilent, 2011 ▶) *T*_min_ = 0.284, *T*_max_ = 0.401
11875 measured reflections
5921 independent reflections
4597 reflections with *I* > 2σ(*I*)
*R*_int_ = 0.056

#### Refinement

*R*[*F* ^2^ > 2σ(*F* ^2^)] = 0.056
*wR*(*F* ^2^) = 0.140
*S* = 1.03
5921 reflections
535 parameters
214 restraints
H-atom parameters constrained
Δρ_max_ = 0.89 e Å^−3^
Δρ_min_ = −0.77 e Å^−3^
Absolute structure: Flack (1983 ▶), 1898 Friedel pairs
Flack parameter: −0.026 (13)

### 

Data collection: *CrysAlis PRO* (Agilent, 2011 ▶); cell refinement: *CrysAlis PRO*; data reduction: *CrysAlis PRO*; program(s) used to solve structure: *SHELXS97* (Sheldrick, 2008 ▶); program(s) used to refine structure: *SHELXL97* (Sheldrick, 2008 ▶); molecular graphics: *ORTEP-3* (Farrugia, 1997 ▶) and *DIAMOND* (Brandenburg, 2006 ▶); software used to prepare material for publication: *publCIF* (Westrip, 2010 ▶).

## Supplementary Material

Crystal structure: contains datablock(s) global, I. DOI: 10.1107/S1600536812011671/hg5191sup1.cif

Structure factors: contains datablock(s) I. DOI: 10.1107/S1600536812011671/hg5191Isup2.hkl

Additional supplementary materials:  crystallographic information; 3D view; checkCIF report

## Figures and Tables

**Table 1:** Selected bond lengths (Å)

Ni—N1	1.930 (7)
Ni—N2	1.939 (7)
Ni—N3	1.929 (7)
Ni—N4	1.929 (7)

**Table 2:** Hydrogen-bond geometry (Å, °)

*D*—H⋯*A*	*D*—H	H⋯*A*	*D*⋯*A*	*D*—H⋯*A*
C19—H19⋯Br4^i^	0.95	2.89	3.728 (8)	148

Symmetry code: (i) 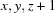.

**Fig. 1.**
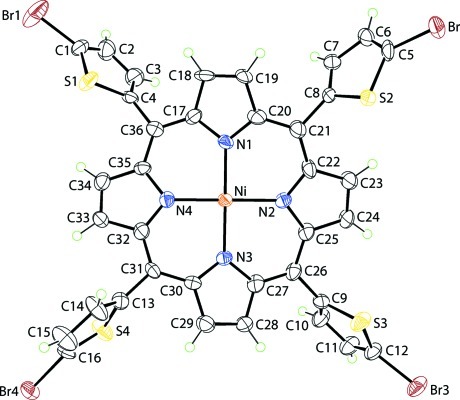
The molecular structure of the title compound showing the atom-labelling scheme and displacement ellipsoids at the 70% probability level. Only the major components of the disorder are shown.

**Fig. 2.**
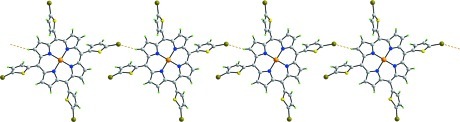
A view of the supramolecular chain along [001] in (I). The C—H···Br interactions are shown as orange dashed lines.

## supplementary crystallographic information

### Comment

Porphyrins with five-membered thienyl rings at the *meso* positions can extend the π-conjugation of the porphyrin core owing to its smaller size and this has led to many investigations on their physiochemical properties (Boyle *et al.*, 2010). They are of interest for their electron and energy transfer properties (Wallin *et al.*, 2006) and because they are capable of growing ultra-thin films (Friedlein *et al.*, 2005). *Meso* tetrathienylporphyrins show interesting electrochemical (Chen *et al.*, 2010), structural (Bhyrappa & Bhavana, 2001; Bhyrappa *et al.*, 2006; Paul-Roth *et al.*, 2008) and photophysical (Rochford *et al.*, 2008) properties. Herein, the synthesis and crystal structure of 5,10,15,20-tetrakis(5-bromothien-2-yl)porphyrinato nickel(II), (I), is reported in continuation of earlier structural studies (Purushothaman *et al.*, 2001).

The Ni^II^ atom in (I), Fig. 1, is in a square-planar geometry defined by four pyrrole-N atoms, Table 1. The coordination geometry resembles that observed in the analogous Zn^II^ complex (Ghazzali *et al.*, 2008). However, in contrast to the literature structure in which the porphyrin molecule (excluding the bromothienyl residues) is essentially planar, there is considerable buckling in (I). This is quantified by the dihedral angles between the N_4_ donor set and the N1–N4-pyrrole rings of 18.8 (3), 18.0 (3), 17.0 (3) and 17.8 (2)°, respectively. This is further quantified in the parameters associated with the six-membered chelate rings. There is a measure of buckling in each of these about an Ni—N bond: NiN1N2 ring [r.m.s. deviation for the six atoms = 0.134 Å with maximum deviations from the least-squares plane = 0.145 (7) Å for the N1 atom and -0.121 (1) Å for the Ni atom], NiN1N4 ring [r.m.s. = 0.124 Å; 0.131 (7) Å (N4) and -0.114 (1) Å (Ni)], NiN2N3 ring [r.m.s. = 0.141 Å; -0.146 (7) Å (N2) and 0.130 (1) Å (Ni)] and NiN3N4 ring [r.m.s. = 0.127 Å; 0.131 (7) Å (N3) and -0.120 (1) Å (Ni)]. The dihedral angles between diagonally opposite six-membered rings are 13.08 (15)° (NiN1N2/NiN3N4) and 13.45 (11)° (NiN2N2/NiN1N4) but each pair is orientated in opposite directions. Each of the bromothienyl rings is twisted out of the plane of the central N_4_ core with the dihedral angles between this and the S1–S4 thienyl rings (major components of the disorder only) being 59.2 (3), 51.7 (2), 71.5 (2) and 74.65 (19)°, respectively.

In the crystal packing, supramolecular chains along [001] are formed through C—H···Br interactions involving the bromide atom of the only non-disordered bromothienyl ring, Fig. 2 and Table 1.

### Experimental

5,10,15,20-Tetrakis[(5-bromo-2-thiophenyl)porphyrin was synthesized as reported in the literature (Friedlein *et al.*, 2005). To 5,10,15,20-Tetrakis[(5-bromo-2-thiophenyl)porphyrin (95 mg, 0.1 mmol) dissolved in dimethylformamide (20 ml), a solution of nickel(II) acetate tetrahydrate (124 mg, 0.5 mmol) in dimethylformamide (5 ml) was added and the resulting solution refluxed for 4 h. After cooling, the solution was diluted with chloroform (100 ml) and washed with water (3×100 ml). The organic layer was dried over anhydrous Na_2_SO_4_. The solvent was removed by distillation and the product was purified by column chromatography using 3:2 chloroform and hexanes as the eluent. Recrystallization was by slow evaporation of a chloroform solution of (I) which yielded purple crystals. Yield: 90%.

### Refinement

Carbon-bound H-atoms were placed in calculated positions [C—H = 0.95 Å, *U*_iso_(H) = 1.2*U*_eq_(C)] and were included in the refinement in the riding model approximation.

Three of the four bromothienyl units are disordered over two positions, the major components being in 0.691 (3), 0.738 (3) and 0.929 (9) fractions. Pairs of 1,2-related distances were restrained to within 0.01 Å and pairs of the1,3-related ones to within 0.02 Å of each other. The *U*_ij_ parameters of the primed carbon atoms were set to those of the unprimed ones; the *U*_ij_ parameters of the bromine and sulfur atoms were not tied but the *U*_ij_ parameters were restrained to be nearly isotropic. For the major components, the atoms were restrained to lie on a plane. Pairs of C_porphyrin_–C_thiophene_ distances were also restrained to within 0.01 Å of each other.

### Crystal data

**Table:**

[Ni(C_36_H_16_Br_4_N_4_S_4_)]	*F*(000) = 3936
*M**_r_* = 1011.12	*D*_x_ = 1.992 Mg m^−^^3^
Orthorhombic, *A**b**a*2	Mo *K*α radiation, λ = 0.71073 Å
Hall symbol: A 2 -2ac	Cell parameters from 3139 reflections
*a* = 21.9367 (9) Å	θ = 2.3–27.5°
*b* = 19.0090 (9) Å	µ = 5.60 mm^−^^1^
*c* = 16.1742 (6) Å	*T* = 100 K
*V* = 6744.6 (5) Å^3^	Prism, purple
*Z* = 8	0.30 × 0.25 × 0.20 mm

### Data collection

**Table:**

Agilent SuperNova Dual diffractometer with an Atlas detector	5921 independent reflections
Radiation source: SuperNova (Mo) X-ray Source	4597 reflections with *I* > 2σ(*I*)
Mirror monochromator	*R*_int_ = 0.056
Detector resolution: 10.4041 pixels mm^-1^	θ_max_ = 27.6°, θ_min_ = 2.3°
ω scan	*h* = −28→20
Absorption correction: multi-scan (*CrysAlis PRO*; Agilent, 2011)	*k* = −24→19
*T*_min_ = 0.284, *T*_max_ = 0.401	*l* = −15→20
11875 measured reflections	

### Refinement

**Table:**

Refinement on *F*^2^	Secondary atom site location: difference Fourier map
Least-squares matrix: full	Hydrogen site location: inferred from neighbouring sites
*R*[*F*^2^ > 2σ(*F*^2^)] = 0.056	H-atom parameters constrained
*wR*(*F*^2^) = 0.140	*w* = 1/[σ^2^(*F*_o_^2^) + (0.0739*P*)^2^] where *P* = (*F*_o_^2^ + 2*F*_c_^2^)/3
*S* = 1.03	(Δ/σ)_max_ = 0.001
5921 reflections	Δρ_max_ = 0.89 e Å^−^^3^
535 parameters	Δρ_min_ = −0.77 e Å^−^^3^
214 restraints	Absolute structure: Flack (1983), 1898 Friedel pairs
Primary atom site location: structure-invariant direct methods	Flack parameter: −0.026 (13)

### Fractional atomic coordinates and isotropic or equivalent isotropic displacement parameters (Å^2^)

**Table:**

	*x*	*y*	*z*	*U*_iso_*/*U*_eq_	Occ. (<1)
Ni	0.93376 (5)	0.79186 (5)	0.49494 (13)	0.0265 (2)	
Br4	0.98930 (5)	0.89722 (6)	−0.04302 (12)	0.0462 (3)	
Br1	1.22918 (9)	1.10301 (11)	0.62570 (19)	0.0835 (8)	0.691 (3)
Br2	0.76615 (7)	0.75530 (8)	1.00080 (15)	0.0555 (5)	0.738 (3)
Br3	0.69378 (6)	0.41916 (10)	0.36601 (18)	0.0477 (7)	0.929 (9)
S1	1.11481 (18)	1.0234 (2)	0.5607 (3)	0.0418 (10)	0.691 (3)
S2	0.80473 (15)	0.76565 (19)	0.8152 (2)	0.0337 (8)	0.738 (3)
S3	0.75144 (18)	0.56085 (19)	0.4230 (3)	0.0451 (10)	0.929 (9)
C1	1.1833 (6)	1.0204 (12)	0.6128 (7)	0.039 (2)	0.691 (3)
C2	1.1970 (7)	0.9570 (8)	0.6395 (7)	0.049 (3)	0.691 (3)
H2	1.2332	0.9466	0.6695	0.059*	0.691 (3)
C3	1.1542 (8)	0.9075 (10)	0.6201 (9)	0.044 (5)	0.691 (3)
H3	1.1575	0.8593	0.6349	0.053*	0.691 (3)
C4	1.1060 (4)	0.9349 (6)	0.5769 (5)	0.028 (3)	0.691 (3)
C5	0.8274 (9)	0.7573 (2)	0.9198 (8)	0.038 (2)	0.738 (3)
C6	0.8860 (6)	0.7533 (4)	0.9284 (7)	0.037 (3)	0.738 (3)
H6	0.9054	0.7486	0.9807	0.044*	0.738 (3)
C7	0.9190 (7)	0.7566 (5)	0.8522 (10)	0.033 (4)	0.738 (3)
H7	0.9621	0.7544	0.8481	0.040*	0.738 (3)
C8	0.8812 (4)	0.7633 (3)	0.7866 (6)	0.023 (2)	0.738 (3)
C9	0.7624 (4)	0.4779 (5)	0.3806 (3)	0.037 (2)	0.929 (9)
C10	0.8204 (4)	0.4649 (5)	0.3627 (5)	0.042 (3)	0.929 (9)
H10	0.8344	0.4221	0.3392	0.050*	0.929 (9)
C11	0.8583 (4)	0.5212 (5)	0.3821 (5)	0.042 (3)	0.929 (9)
H11	0.9010	0.5201	0.3728	0.050*	0.929 (9)
C12	0.8295 (4)	0.5778 (5)	0.4154 (4)	0.034 (3)	0.929 (9)
Br1'	1.26243 (13)	1.04721 (18)	0.6642 (2)	0.0448 (11)	0.309 (3)
Br2'	0.8321 (2)	0.7373 (3)	1.0322 (3)	0.0637 (16)	0.262 (3)
Br3'	0.6913 (13)	0.4343 (17)	0.329 (3)	0.082 (10)	0.071 (9)
S1'	1.1596 (4)	0.9388 (5)	0.6245 (6)	0.034 (2)	0.309 (3)
S2'	0.8946 (5)	0.7495 (7)	0.8595 (7)	0.038 (3)	0.262 (3)
S3'	0.746 (2)	0.5740 (17)	0.395 (3)	0.034 (13)	0.071 (9)
C1'	1.1861 (11)	1.0237 (15)	0.618 (2)	0.039 (2)	0.309
C2'	1.1515 (11)	1.0620 (12)	0.5691 (18)	0.049 (3)	0.309
H2'	1.1606	1.1095	0.5556	0.059*	0.309 (3)
C3'	1.1012 (15)	1.0277 (15)	0.539 (3)	0.044 (5)	0.309
H3'	1.0719	1.0496	0.5047	0.053*	0.309 (3)
C4'	1.0968 (9)	0.9583 (9)	0.5643 (18)	0.028 (3)	0.309
C5'	0.8271 (14)	0.7572 (19)	0.9185 (10)	0.038 (2)	0.262
C6'	0.7797 (8)	0.7699 (18)	0.8732 (11)	0.037 (3)	0.262
H6'	0.7396	0.7735	0.8951	0.044*	0.262 (3)
C7'	0.7930 (11)	0.778 (2)	0.7870 (13)	0.033 (4)	0.262
H7'	0.7632	0.7872	0.7458	0.040*	0.262 (3)
C8'	0.8534 (7)	0.7712 (17)	0.7713 (8)	0.023 (2)	0.262
C9'	0.756 (2)	0.484 (2)	0.380 (6)	0.037 (2)	0.071
C10'	0.813 (3)	0.463 (3)	0.392 (9)	0.042 (3)	0.071
H10B	0.8255	0.4154	0.3891	0.050*	0.071 (9)
C11'	0.851 (2)	0.519 (4)	0.409 (9)	0.042 (3)	0.071
H11B	0.8946	0.5152	0.4084	0.050*	0.071 (9)
C12'	0.822 (3)	0.5800 (19)	0.428 (8)	0.034 (3)	0.071
S4	0.97840 (12)	0.83684 (15)	0.13430 (18)	0.0472 (6)	
N1	0.9641 (3)	0.8293 (4)	0.5979 (4)	0.0302 (16)	
N2	0.8858 (3)	0.7234 (4)	0.5555 (4)	0.0321 (17)	
N3	0.9020 (3)	0.7566 (4)	0.3917 (4)	0.0285 (16)	
N4	0.9833 (3)	0.8581 (4)	0.4344 (4)	0.0281 (15)	
C13	0.9590 (4)	0.8994 (6)	0.0664 (5)	0.037 (2)	
C14	0.9252 (5)	0.9510 (7)	0.0986 (6)	0.057 (3)	
H14	0.9119	0.9914	0.0691	0.068*	
C15	0.9120 (5)	0.9373 (6)	0.1823 (6)	0.047 (3)	
H15	0.8877	0.9677	0.2153	0.056*	
C16	0.9369 (4)	0.8766 (5)	0.2120 (5)	0.033 (2)	
C17	1.0119 (4)	0.8756 (5)	0.6093 (5)	0.031 (2)	
C18	1.0132 (4)	0.8999 (5)	0.6927 (5)	0.038 (2)	
H18	1.0416	0.9318	0.7163	0.045*	
C19	0.9660 (4)	0.8683 (5)	0.7312 (5)	0.036 (2)	
H19	0.9538	0.8756	0.7869	0.043*	
C20	0.9382 (4)	0.8227 (5)	0.6744 (5)	0.034 (2)	
C21	0.8944 (4)	0.7714 (5)	0.6948 (6)	0.040 (2)	
C22	0.8749 (4)	0.7213 (5)	0.6384 (5)	0.037 (2)	
C23	0.8442 (5)	0.6573 (5)	0.6606 (6)	0.046 (2)	
H23	0.8306	0.6439	0.7142	0.055*	
C24	0.8385 (5)	0.6202 (5)	0.5907 (6)	0.046 (3)	
H24	0.8218	0.5742	0.5859	0.055*	
C25	0.8622 (4)	0.6618 (4)	0.5242 (5)	0.0327 (19)	
C26	0.8555 (4)	0.6462 (5)	0.4399 (6)	0.034 (2)	
C27	0.8693 (4)	0.6955 (5)	0.3789 (6)	0.034 (2)	
C28	0.8496 (4)	0.6909 (5)	0.2965 (5)	0.036 (2)	
H28	0.8275	0.6531	0.2722	0.043*	
C29	0.8681 (4)	0.7508 (5)	0.2581 (6)	0.037 (2)	
H29	0.8594	0.7641	0.2027	0.045*	
C30	0.9027 (3)	0.7897 (4)	0.3163 (5)	0.0237 (17)	
C31	0.9381 (4)	0.8475 (5)	0.2967 (5)	0.0278 (18)	
C32	0.9796 (4)	0.8749 (4)	0.3524 (5)	0.0318 (19)	
C33	1.0303 (4)	0.9195 (5)	0.3295 (5)	0.033 (2)	
H33	1.0377	0.9390	0.2763	0.040*	
C34	1.0651 (4)	0.9283 (5)	0.3968 (6)	0.036 (2)	
H34	1.1030	0.9522	0.3997	0.044*	
C35	1.0337 (3)	0.8946 (4)	0.4634 (5)	0.0285 (18)	
C36	1.0484 (4)	0.9021 (5)	0.5462 (5)	0.035 (2)	

### Atomic displacement parameters (Å^2^)

**Table:**

	*U*^11^	*U*^22^	*U*^33^	*U*^12^	*U*^13^	*U*^23^
Ni	0.0393 (5)	0.0181 (5)	0.0220 (4)	−0.0025 (4)	0.0002 (5)	−0.0026 (5)
Br4	0.0635 (6)	0.0480 (7)	0.0269 (4)	−0.0073 (5)	0.0044 (5)	0.0000 (5)
Br1	0.0654 (12)	0.0559 (13)	0.1291 (19)	−0.0263 (9)	−0.0319 (12)	−0.0049 (13)
Br2	0.0674 (10)	0.0571 (10)	0.0418 (8)	−0.0203 (7)	0.0249 (8)	−0.0030 (7)
Br3	0.0402 (6)	0.0393 (8)	0.0637 (14)	−0.0117 (5)	0.0056 (7)	−0.0212 (8)
S1	0.044 (2)	0.0252 (19)	0.057 (3)	−0.0061 (15)	−0.0136 (17)	−0.0011 (17)
S2	0.0391 (16)	0.033 (2)	0.0289 (16)	−0.0082 (14)	0.0072 (14)	−0.0059 (15)
S3	0.0436 (16)	0.0361 (17)	0.056 (2)	−0.0079 (14)	0.0078 (18)	−0.0208 (17)
C1	0.043 (4)	0.034 (5)	0.040 (5)	−0.011 (4)	−0.009 (4)	−0.003 (4)
C2	0.042 (6)	0.054 (8)	0.050 (7)	−0.008 (6)	−0.001 (6)	0.008 (6)
C3	0.051 (7)	0.040 (8)	0.042 (7)	−0.002 (7)	−0.008 (6)	0.005 (7)
C4	0.038 (5)	0.030 (7)	0.017 (5)	0.002 (5)	−0.006 (4)	0.000 (5)
C5	0.050 (5)	0.023 (5)	0.041 (5)	−0.009 (4)	0.018 (4)	0.006 (4)
C6	0.055 (6)	0.026 (6)	0.029 (5)	−0.003 (5)	0.004 (5)	0.006 (4)
C7	0.040 (7)	0.028 (6)	0.032 (7)	−0.001 (6)	0.005 (6)	−0.007 (5)
C8	0.035 (6)	0.010 (4)	0.025 (5)	−0.001 (5)	0.004 (5)	0.000 (4)
C9	0.037 (4)	0.031 (5)	0.043 (5)	−0.007 (4)	0.004 (4)	−0.012 (4)
C10	0.042 (5)	0.029 (5)	0.055 (7)	0.000 (4)	0.001 (5)	−0.013 (5)
C11	0.041 (5)	0.034 (5)	0.050 (7)	−0.008 (4)	0.002 (4)	−0.008 (5)
C12	0.047 (5)	0.028 (5)	0.025 (5)	−0.005 (4)	0.006 (4)	−0.009 (4)
Br1'	0.0336 (16)	0.045 (2)	0.056 (2)	−0.0027 (13)	−0.0114 (13)	−0.0138 (16)
Br2'	0.098 (4)	0.062 (3)	0.031 (2)	0.010 (3)	0.006 (2)	0.0104 (19)
Br3'	0.087 (12)	0.078 (12)	0.081 (13)	0.008 (8)	0.010 (9)	−0.002 (9)
S1'	0.040 (4)	0.023 (4)	0.038 (4)	−0.004 (4)	−0.009 (3)	0.013 (4)
S2'	0.040 (6)	0.046 (6)	0.028 (5)	0.009 (4)	−0.010 (5)	0.005 (4)
S3'	0.039 (15)	0.036 (15)	0.027 (15)	0.000 (9)	0.006 (9)	−0.003 (9)
C1'	0.043 (4)	0.034 (5)	0.040 (5)	−0.011 (4)	−0.009 (4)	−0.003 (4)
C2'	0.042 (6)	0.054 (8)	0.050 (7)	−0.008 (6)	−0.001 (6)	0.008 (6)
C3'	0.051 (7)	0.040 (8)	0.042 (7)	−0.002 (7)	−0.008 (6)	0.005 (7)
C4'	0.038 (5)	0.030 (7)	0.017 (5)	0.002 (5)	−0.006 (4)	0.000 (5)
C5'	0.050 (5)	0.023 (5)	0.041 (5)	−0.009 (4)	0.018 (4)	0.006 (4)
C6'	0.055 (6)	0.026 (6)	0.029 (5)	−0.003 (5)	0.004 (5)	0.006 (4)
C7'	0.040 (7)	0.028 (6)	0.032 (7)	−0.001 (6)	0.005 (6)	−0.007 (5)
C8'	0.035 (6)	0.010 (4)	0.025 (5)	−0.001 (5)	0.004 (5)	0.000 (4)
C9'	0.037 (4)	0.031 (5)	0.043 (5)	−0.007 (4)	0.004 (4)	−0.012 (4)
C10'	0.042 (5)	0.029 (5)	0.055 (7)	0.000 (4)	0.001 (5)	−0.013 (5)
C11'	0.041 (5)	0.034 (5)	0.050 (7)	−0.008 (4)	0.002 (4)	−0.008 (5)
C12'	0.047 (5)	0.028 (5)	0.025 (5)	−0.005 (4)	0.006 (4)	−0.009 (4)
S4	0.0735 (16)	0.0387 (15)	0.0293 (12)	0.0169 (13)	0.0105 (12)	0.0026 (11)
N1	0.049 (4)	0.022 (4)	0.020 (3)	−0.003 (3)	0.003 (3)	−0.002 (3)
N2	0.045 (4)	0.019 (4)	0.032 (4)	−0.005 (3)	0.005 (3)	−0.008 (3)
N3	0.036 (4)	0.023 (4)	0.027 (4)	−0.003 (3)	0.003 (3)	−0.007 (3)
N4	0.032 (3)	0.022 (4)	0.030 (4)	0.000 (3)	0.005 (3)	−0.002 (3)
C13	0.045 (5)	0.054 (7)	0.012 (4)	−0.008 (5)	−0.001 (4)	−0.003 (4)
C14	0.067 (7)	0.064 (8)	0.040 (6)	0.026 (6)	0.007 (5)	0.011 (5)
C15	0.064 (6)	0.045 (6)	0.030 (5)	0.030 (5)	0.019 (5)	0.008 (5)
C16	0.040 (4)	0.029 (5)	0.029 (4)	0.000 (4)	0.009 (4)	−0.006 (4)
C17	0.034 (4)	0.025 (5)	0.035 (5)	−0.005 (4)	−0.011 (4)	0.002 (4)
C18	0.050 (5)	0.042 (6)	0.021 (4)	−0.002 (4)	−0.013 (4)	−0.010 (4)
C19	0.063 (6)	0.024 (5)	0.021 (4)	−0.004 (4)	0.010 (4)	−0.009 (4)
C20	0.050 (5)	0.023 (5)	0.029 (4)	0.006 (4)	0.002 (4)	−0.007 (4)
C21	0.056 (6)	0.031 (6)	0.032 (5)	−0.007 (5)	0.010 (5)	−0.003 (4)
C22	0.055 (5)	0.022 (5)	0.033 (5)	−0.003 (4)	0.016 (4)	0.002 (4)
C23	0.077 (7)	0.027 (5)	0.033 (5)	−0.013 (5)	0.008 (5)	−0.001 (4)
C24	0.074 (7)	0.021 (5)	0.042 (5)	−0.020 (5)	0.008 (5)	0.000 (4)
C25	0.044 (4)	0.017 (4)	0.037 (5)	−0.001 (4)	0.008 (4)	−0.004 (4)
C26	0.038 (4)	0.023 (5)	0.041 (5)	−0.004 (4)	0.008 (4)	−0.006 (4)
C27	0.041 (4)	0.021 (5)	0.038 (5)	0.002 (4)	0.007 (4)	−0.006 (4)
C28	0.049 (5)	0.028 (5)	0.030 (5)	−0.008 (4)	−0.001 (4)	−0.004 (4)
C29	0.044 (5)	0.037 (6)	0.031 (4)	0.001 (4)	−0.004 (4)	0.002 (4)
C30	0.027 (4)	0.023 (5)	0.022 (4)	0.008 (3)	−0.002 (3)	−0.004 (3)
C31	0.038 (4)	0.025 (5)	0.020 (4)	0.004 (4)	0.003 (4)	0.004 (3)
C32	0.045 (5)	0.019 (5)	0.032 (5)	0.000 (4)	0.004 (4)	−0.001 (4)
C33	0.043 (5)	0.033 (5)	0.024 (4)	−0.003 (4)	0.002 (4)	0.002 (4)
C34	0.041 (5)	0.028 (5)	0.040 (5)	−0.005 (4)	0.005 (4)	−0.002 (4)
C35	0.032 (4)	0.027 (5)	0.026 (4)	0.001 (3)	−0.009 (4)	−0.001 (4)
C36	0.040 (5)	0.038 (6)	0.028 (4)	−0.003 (4)	−0.005 (4)	0.002 (4)

### Geometric parameters (Å, º)

**Table:**

Ni—N1	1.930 (7)	C8'—C21	1.530 (15)
Ni—N2	1.939 (7)	C9'—C10'	1.331 (15)
Ni—N3	1.929 (7)	C10'—C11'	1.392 (16)
Ni—N4	1.929 (7)	C10'—H10B	0.9500
Br4—C13	1.891 (8)	C11'—C12'	1.360 (16)
Br1—C1	1.877 (14)	C11'—H11B	0.9500
Br2—C5	1.878 (12)	C12'—C26	1.472 (15)
Br3—C9	1.889 (8)	S4—C13	1.674 (10)
S1—C4	1.712 (12)	S4—C16	1.726 (9)
S1—C1	1.724 (18)	N1—C20	1.368 (11)
S2—C8	1.741 (10)	N1—C17	1.379 (11)
S2—C5	1.770 (16)	N2—C22	1.363 (11)
S3—C9	1.736 (10)	N2—C25	1.376 (10)
S3—C12	1.746 (10)	N3—C30	1.372 (10)
C1—C2	1.32 (3)	N3—C27	1.380 (11)
C2—C3	1.37 (2)	N4—C32	1.367 (11)
C2—H2	0.9500	N4—C35	1.386 (10)
C3—C4	1.37 (2)	C13—C14	1.334 (14)
C3—H3	0.9500	C14—C15	1.409 (13)
C4—C36	1.493 (13)	C14—H14	0.9500
C5—C6	1.30 (2)	C15—C16	1.364 (13)
C6—C7	1.43 (2)	C15—H15	0.9500
C6—H6	0.9500	C16—C31	1.477 (12)
C7—C8	1.35 (2)	C17—C36	1.394 (12)
C7—H7	0.9500	C17—C18	1.425 (12)
C8—C21	1.521 (13)	C18—C19	1.350 (13)
C9—C10	1.328 (12)	C18—H18	0.9500
C10—C11	1.390 (13)	C19—C20	1.402 (12)
C10—H10	0.9500	C19—H19	0.9500
C11—C12	1.359 (13)	C20—C21	1.408 (13)
C11—H11	0.9500	C21—C22	1.386 (13)
C12—C26	1.476 (12)	C22—C23	1.438 (13)
Br1'—C1'	1.890 (16)	C23—C24	1.339 (13)
Br2'—C5'	1.879 (14)	C23—H23	0.9500
Br3'—C9'	1.888 (13)	C24—C25	1.432 (12)
S1'—C1'	1.72 (2)	C24—H24	0.9500
S1'—C4'	1.727 (14)	C25—C26	1.404 (12)
S2'—C8'	1.738 (13)	C26—C27	1.393 (13)
S2'—C5'	1.769 (19)	C27—C28	1.405 (12)
S3'—C9'	1.738 (14)	C28—C29	1.358 (12)
S3'—C12'	1.747 (14)	C28—H28	0.9500
C1'—C2'	1.31 (3)	C29—C30	1.419 (11)
C2'—C3'	1.37 (2)	C29—H29	0.9500
C2'—H2'	0.9500	C30—C31	1.382 (12)
C3'—C4'	1.38 (2)	C31—C32	1.383 (12)
C3'—H3'	0.9500	C32—C33	1.448 (12)
C4'—C36	1.532 (14)	C33—C34	1.339 (12)
C5'—C6'	1.29 (3)	C33—H33	0.9500
C6'—C7'	1.43 (2)	C34—C35	1.430 (12)
C6'—H6'	0.9500	C34—H34	0.9500
C7'—C8'	1.36 (2)	C35—C36	1.386 (12)
C7'—H7'	0.9500		
			
N3—Ni—N4	89.5 (3)	C22—N2—C25	105.8 (7)
N3—Ni—N1	178.4 (3)	C22—N2—Ni	127.6 (6)
N4—Ni—N1	90.1 (3)	C25—N2—Ni	126.1 (5)
N3—Ni—N2	90.4 (3)	C30—N3—C27	104.9 (7)
N4—Ni—N2	178.4 (3)	C30—N3—Ni	127.3 (5)
N1—Ni—N2	90.0 (3)	C27—N3—Ni	127.6 (6)
C4—S1—C1	89.5 (8)	C32—N4—C35	104.9 (7)
C8—S2—C5	88.9 (7)	C32—N4—Ni	127.7 (6)
C9—S3—C12	90.2 (4)	C35—N4—Ni	127.2 (5)
C2—C1—S1	113.0 (11)	C14—C13—S4	114.0 (7)
C2—C1—Br1	127.4 (13)	C14—C13—Br4	125.2 (8)
S1—C1—Br1	119.6 (15)	S4—C13—Br4	120.6 (6)
C1—C2—C3	113.4 (15)	C13—C14—C15	110.7 (9)
C1—C2—H2	123.3	C13—C14—H14	124.6
C3—C2—H2	123.3	C15—C14—H14	124.6
C2—C3—C4	112.7 (15)	C16—C15—C14	114.4 (9)
C2—C3—H3	123.6	C16—C15—H15	122.8
C4—C3—H3	123.6	C14—C15—H15	122.8
C3—C4—C36	131.6 (12)	C15—C16—C31	130.6 (8)
C3—C4—S1	111.3 (10)	C15—C16—S4	108.9 (7)
C36—C4—S1	117.0 (8)	C31—C16—S4	120.1 (7)
C6—C5—S2	112.8 (9)	N1—C17—C36	124.7 (8)
C6—C5—Br2	129.3 (12)	N1—C17—C18	110.4 (7)
S2—C5—Br2	117.9 (12)	C36—C17—C18	124.3 (8)
C5—C6—C7	113.9 (12)	C19—C18—C17	106.1 (8)
C5—C6—H6	123.0	C19—C18—H18	127.0
C7—C6—H6	123.0	C17—C18—H18	127.0
C8—C7—C6	111.8 (13)	C18—C19—C20	107.8 (7)
C8—C7—H7	124.1	C18—C19—H19	126.1
C6—C7—H7	124.1	C20—C19—H19	126.1
C7—C8—C21	131.3 (10)	N1—C20—C19	110.8 (8)
C7—C8—S2	112.6 (9)	N1—C20—C21	124.0 (8)
C21—C8—S2	116.2 (7)	C19—C20—C21	124.9 (8)
C10—C9—S3	112.8 (7)	C22—C21—C20	122.2 (8)
C10—C9—Br3	128.8 (8)	C22—C21—C8	120.9 (8)
S3—C9—Br3	118.4 (5)	C20—C21—C8	115.4 (8)
C9—C10—C11	112.4 (9)	C22—C21—C8'	110.4 (14)
C9—C10—H10	123.8	C20—C21—C8'	126.3 (15)
C11—C10—H10	123.8	N2—C22—C21	125.0 (8)
C12—C11—C10	114.9 (9)	N2—C22—C23	110.6 (8)
C12—C11—H11	122.6	C21—C22—C23	124.2 (8)
C10—C11—H11	122.6	C24—C23—C22	106.2 (8)
C11—C12—C26	128.7 (9)	C24—C23—H23	126.9
C11—C12—S3	109.7 (7)	C22—C23—H23	126.9
C26—C12—S3	121.6 (7)	C23—C24—C25	108.0 (8)
C1'—S1'—C4'	91.9 (10)	C23—C24—H24	126.0
C8'—S2'—C5'	89.3 (9)	C25—C24—H24	126.0
C9'—S3'—C12'	89.7 (8)	N2—C25—C26	125.1 (8)
C2'—C1'—S1'	111.3 (12)	N2—C25—C24	109.3 (7)
C2'—C1'—Br1'	128.2 (17)	C26—C25—C24	125.1 (8)
S1'—C1'—Br1'	119.8 (19)	C27—C26—C25	121.6 (8)
C1'—C2'—C3'	114.3 (17)	C27—C26—C12'	126 (5)
C1'—C2'—H2'	122.9	C25—C26—C12'	111 (5)
C3'—C2'—H2'	122.9	C27—C26—C12	119.1 (8)
C2'—C3'—C4'	114.2 (18)	C25—C26—C12	119.2 (8)
C2'—C3'—H3'	122.9	N3—C27—C26	124.9 (8)
C4'—C3'—H3'	122.9	N3—C27—C28	110.8 (8)
C3'—C4'—C36	131.1 (15)	C26—C27—C28	124.2 (8)
C3'—C4'—S1'	108.2 (12)	C29—C28—C27	106.8 (8)
C36—C4'—S1'	120.7 (11)	C29—C28—H28	126.6
C6'—C5'—S2'	112.5 (11)	C27—C28—H28	126.6
C6'—C5'—Br2'	129.7 (16)	C28—C29—C30	107.1 (8)
S2'—C5'—Br2'	117.5 (16)	C28—C29—H29	126.5
C5'—C6'—C7'	114.0 (15)	C30—C29—H29	126.5
C5'—C6'—H6'	123.0	N3—C30—C31	125.1 (7)
C7'—C6'—H6'	123.0	N3—C30—C29	110.2 (7)
C8'—C7'—C6'	111.8 (16)	C31—C30—C29	124.3 (7)
C8'—C7'—H7'	124.1	C30—C31—C32	121.3 (7)
C6'—C7'—H7'	124.1	C30—C31—C16	120.1 (8)
C7'—C8'—C21	136.5 (14)	C32—C31—C16	118.4 (7)
C7'—C8'—S2'	112.2 (12)	N4—C32—C31	125.7 (7)
C21—C8'—S2'	111.1 (9)	N4—C32—C33	109.9 (7)
C10'—C9'—S3'	112.8 (11)	C31—C32—C33	124.0 (8)
C10'—C9'—Br3'	128.0 (19)	C34—C33—C32	107.6 (8)
S3'—C9'—Br3'	117.8 (15)	C34—C33—H33	126.2
C9'—C10'—C11'	111.9 (15)	C32—C33—H33	126.2
C9'—C10'—H10B	124.0	C33—C34—C35	106.4 (8)
C11'—C10'—H10B	124.0	C33—C34—H34	126.8
C12'—C11'—C10'	113.9 (18)	C35—C34—H34	126.8
C12'—C11'—H11B	123.0	C36—C35—N4	124.4 (8)
C10'—C11'—H11B	123.0	C36—C35—C34	124.7 (8)
C11'—C12'—C26	121 (6)	N4—C35—C34	110.8 (7)
C11'—C12'—S3'	109.3 (15)	C35—C36—C17	122.4 (8)
C26—C12'—S3'	125 (3)	C35—C36—C4	124.3 (8)
C13—S4—C16	91.9 (5)	C17—C36—C4	113.2 (7)
C20—N1—C17	104.6 (7)	C35—C36—C4'	114.8 (13)
C20—N1—Ni	127.1 (6)	C17—C36—C4'	120.7 (14)
C17—N1—Ni	127.8 (6)		
			
C4—S1—C1—C2	−0.1 (2)	C7'—C8'—C21—C8	−175 (7)
C4—S1—C1—Br1	179.96 (12)	S2'—C8'—C21—C8	−1.4 (14)
S1—C1—C2—C3	0.2 (3)	C25—N2—C22—C21	176.0 (9)
Br1—C1—C2—C3	−179.9 (2)	Ni—N2—C22—C21	3.6 (14)
C1—C2—C3—C4	−0.2 (5)	C25—N2—C22—C23	0.2 (11)
C2—C3—C4—C36	−177.2 (7)	Ni—N2—C22—C23	−172.2 (7)
C2—C3—C4—S1	0.1 (4)	C20—C21—C22—N2	−13.2 (16)
C1—S1—C4—C3	0.0 (3)	C8—C21—C22—N2	−178.5 (9)
C1—S1—C4—C36	177.7 (6)	C8'—C21—C22—N2	155.6 (11)
C8—S2—C5—C6	−0.1 (2)	C20—C21—C22—C23	162.0 (10)
C8—S2—C5—Br2	179.95 (11)	C8—C21—C22—C23	−3.2 (15)
S2—C5—C6—C7	0.1 (3)	C8'—C21—C22—C23	−29.2 (15)
Br2—C5—C6—C7	−179.99 (18)	N2—C22—C23—C24	1.9 (12)
C5—C6—C7—C8	0.0 (4)	C21—C22—C23—C24	−173.9 (10)
C6—C7—C8—C21	177.5 (7)	C22—C23—C24—C25	−3.2 (12)
C6—C7—C8—S2	−0.1 (4)	C22—N2—C25—C26	170.4 (9)
C5—S2—C8—C7	0.1 (3)	Ni—N2—C25—C26	−17.0 (13)
C5—S2—C8—C21	−177.9 (5)	C22—N2—C25—C24	−2.2 (10)
C12—S3—C9—C10	0.1 (2)	Ni—N2—C25—C24	170.4 (7)
C12—S3—C9—Br3	180.00 (12)	C23—C24—C25—N2	3.5 (12)
S3—C9—C10—C11	−0.2 (3)	C23—C24—C25—C26	−169.1 (10)
Br3—C9—C10—C11	179.91 (18)	N2—C25—C26—C27	−4.2 (14)
C9—C10—C11—C12	0.2 (4)	C24—C25—C26—C27	167.4 (9)
C10—C11—C12—C26	−177.6 (6)	N2—C25—C26—C12'	−173 (3)
C10—C11—C12—S3	−0.2 (4)	C24—C25—C26—C12'	−1 (3)
C9—S3—C12—C11	0.1 (3)	N2—C25—C26—C12	180.0 (8)
C9—S3—C12—C26	177.7 (6)	C24—C25—C26—C12	−8.5 (14)
C4'—S1'—C1'—C2'	−4 (3)	C11'—C12'—C26—C27	91 (8)
C4'—S1'—C1'—Br1'	−175.1 (19)	S3'—C12'—C26—C27	−63 (11)
S1'—C1'—C2'—C3'	4 (4)	C11'—C12'—C26—C25	−101 (9)
Br1'—C1'—C2'—C3'	174 (3)	S3'—C12'—C26—C25	105 (8)
C1'—C2'—C3'—C4'	−2 (6)	C11'—C12'—C26—C12	41 (14)
C2'—C3'—C4'—C36	179 (3)	S3'—C12'—C26—C12	−113 (22)
C2'—C3'—C4'—S1'	−1 (5)	C11—C12—C26—C27	77.1 (10)
C1'—S1'—C4'—C3'	3 (3)	S3—C12—C26—C27	−100.1 (8)
C1'—S1'—C4'—C36	−178 (2)	C11—C12—C26—C25	−107.0 (8)
C8'—S2'—C5'—C6'	4 (3)	S3—C12—C26—C25	75.8 (9)
C8'—S2'—C5'—Br2'	178 (2)	C11—C12—C26—C12'	−148 (16)
S2'—C5'—C6'—C7'	−3 (5)	S3—C12—C26—C12'	35 (16)
Br2'—C5'—C6'—C7'	−176 (3)	C30—N3—C27—C26	−178.1 (8)
C5'—C6'—C7'—C8'	−1 (5)	Ni—N3—C27—C26	−2.8 (12)
C6'—C7'—C8'—C21	177 (3)	C30—N3—C27—C28	−0.4 (9)
C6'—C7'—C8'—S2'	4 (4)	Ni—N3—C27—C28	174.9 (6)
C5'—S2'—C8'—C7'	−4 (3)	C25—C26—C27—N3	14.4 (13)
C5'—S2'—C8'—C21	−179 (2)	C12'—C26—C27—N3	−179 (4)
C12'—S3'—C9'—C10'	−5 (9)	C12—C26—C27—N3	−169.7 (8)
C12'—S3'—C9'—Br3'	−173 (7)	C25—C26—C27—C28	−163.0 (9)
S3'—C9'—C10'—C11'	−4 (12)	C12'—C26—C27—C28	4 (4)
Br3'—C9'—C10'—C11'	163 (10)	C12—C26—C27—C28	12.9 (13)
C9'—C10'—C11'—C12'	13 (13)	N3—C27—C28—C29	−2.2 (10)
C10'—C11'—C12'—C26	−174 (12)	C26—C27—C28—C29	175.5 (8)
C10'—C11'—C12'—S3'	−17 (10)	C27—C28—C29—C30	3.8 (10)
C9'—S3'—C12'—C11'	12 (8)	C27—N3—C30—C31	−169.6 (8)
C9'—S3'—C12'—C26	168 (10)	Ni—N3—C30—C31	15.1 (11)
N4—Ni—N1—C20	160.5 (8)	C27—N3—C30—C29	2.8 (9)
N2—Ni—N1—C20	−21.1 (8)	Ni—N3—C30—C29	−172.5 (6)
N4—Ni—N1—C17	−10.7 (7)	C28—C29—C30—N3	−4.3 (10)
N2—Ni—N1—C17	167.7 (8)	C28—C29—C30—C31	168.2 (8)
N3—Ni—N2—C22	−167.9 (8)	N3—C30—C31—C32	3.8 (13)
N1—Ni—N2—C22	10.6 (8)	C29—C30—C31—C32	−167.6 (8)
N3—Ni—N2—C25	21.1 (8)	N3—C30—C31—C16	177.3 (7)
N1—Ni—N2—C25	−160.4 (8)	C29—C30—C31—C16	6.0 (12)
N4—Ni—N3—C30	−19.3 (7)	C15—C16—C31—C30	107.8 (12)
N2—Ni—N3—C30	162.3 (7)	S4—C16—C31—C30	−79.6 (10)
N4—Ni—N3—C27	166.5 (7)	C15—C16—C31—C32	−78.4 (13)
N2—Ni—N3—C27	−12.0 (7)	S4—C16—C31—C32	94.2 (9)
N3—Ni—N4—C32	11.8 (7)	C35—N4—C32—C31	176.1 (8)
N1—Ni—N4—C32	−166.7 (7)	Ni—N4—C32—C31	0.9 (12)
N3—Ni—N4—C35	−162.4 (7)	C35—N4—C32—C33	3.1 (9)
N1—Ni—N4—C35	19.2 (7)	Ni—N4—C32—C33	−172.1 (6)
C16—S4—C13—C14	2.9 (9)	C30—C31—C32—N4	−12.0 (13)
C16—S4—C13—Br4	178.3 (6)	C16—C31—C32—N4	174.3 (8)
S4—C13—C14—C15	−2.8 (13)	C30—C31—C32—C33	160.1 (8)
Br4—C13—C14—C15	−178.1 (8)	C16—C31—C32—C33	−13.6 (13)
C13—C14—C15—C16	1.2 (15)	N4—C32—C33—C34	1.2 (10)
C14—C15—C16—C31	174.1 (10)	C31—C32—C33—C34	−171.9 (8)
C14—C15—C16—S4	0.8 (13)	C32—C33—C34—C35	−4.9 (10)
C13—S4—C16—C15	−2.0 (8)	C32—N4—C35—C36	168.8 (9)
C13—S4—C16—C31	−176.1 (7)	Ni—N4—C35—C36	−16.0 (12)
C20—N1—C17—C36	−174.7 (9)	C32—N4—C35—C34	−6.2 (9)
Ni—N1—C17—C36	−2.0 (13)	Ni—N4—C35—C34	169.0 (6)
C20—N1—C17—C18	−2.8 (10)	C33—C34—C35—C36	−167.9 (9)
Ni—N1—C17—C18	169.9 (6)	C33—C34—C35—N4	7.1 (10)
N1—C17—C18—C19	−0.2 (11)	N4—C35—C36—C17	−2.7 (15)
C36—C17—C18—C19	171.8 (9)	C34—C35—C36—C17	171.6 (9)
C17—C18—C19—C20	3.0 (11)	N4—C35—C36—C4	173.3 (8)
C17—N1—C20—C19	4.8 (10)	C34—C35—C36—C4	−12.3 (15)
Ni—N1—C20—C19	−168.0 (6)	N4—C35—C36—C4'	−166.3 (12)
C17—N1—C20—C21	−168.6 (9)	C34—C35—C36—C4'	8.0 (16)
Ni—N1—C20—C21	18.6 (13)	N1—C17—C36—C35	11.9 (15)
C18—C19—C20—N1	−5.1 (11)	C18—C17—C36—C35	−158.9 (9)
C18—C19—C20—C21	168.3 (9)	N1—C17—C36—C4	−164.5 (9)
N1—C20—C21—C22	1.9 (15)	C18—C17—C36—C4	24.7 (13)
C19—C20—C21—C22	−170.6 (9)	N1—C17—C36—C4'	174.5 (11)
N1—C20—C21—C8	167.9 (8)	C18—C17—C36—C4'	3.7 (16)
C19—C20—C21—C8	−4.6 (14)	C3—C4—C36—C35	−114.9 (10)
N1—C20—C21—C8'	−165.1 (11)	S1—C4—C36—C35	68.0 (11)
C19—C20—C21—C8'	22.4 (17)	C3—C4—C36—C17	61.5 (10)
C7—C8—C21—C22	117.9 (9)	S1—C4—C36—C17	−115.6 (8)
S2—C8—C21—C22	−64.5 (10)	C3—C4—C36—C4'	178 (4)
C7—C8—C21—C20	−48.3 (11)	S1—C4—C36—C4'	1 (4)
S2—C8—C21—C20	129.3 (7)	C3'—C4'—C36—C35	50 (4)
C7—C8—C21—C8'	−170 (3)	S1'—C4'—C36—C35	−129.4 (18)
S2—C8—C21—C8'	7 (3)	C3'—C4'—C36—C17	−114 (4)
C7'—C8'—C21—C22	−56 (5)	S1'—C4'—C36—C17	67 (2)
S2'—C8'—C21—C22	118.1 (17)	C3'—C4'—C36—C4	173 (7)
C7'—C8'—C21—C20	113 (4)	S1'—C4'—C36—C4	−6 (2)
S2'—C8'—C21—C20	−74 (2)		

### Hydrogen-bond geometry (Å, º)

**Table:**

*D*—H···*A*	*D*—H	H···*A*	*D*···*A*	*D*—H···*A*
C19—H19···Br4^i^	0.95	2.89	3.728 (8)	148

Symmetry code: (i) *x*, *y*, *z*+1.
